# Preterm infants at low risk for early-onset sepsis differ in early fecal microbiome assembly

**DOI:** 10.1080/19490976.2022.2154091

**Published:** 2022-12-06

**Authors:** Sagori Mukhopadhyay, Jung-Jin Lee, Erica Hartman, Emily Woodford, Miren B. Dhudasia, Lisa M. Mattei, Scott G. Daniel, Kelly C. Wade, Mark A. Underwood, Kyle Bittinger

**Affiliations:** a Division of Neonatology, Children’s Hospital of Philadelphia, Philadelphia, Pennsylvania, United States; b Department of Pediatrics, University of Pennsylvania Perelman School of Medicine, Philadelphia, Pennsylvania, United States; c Center for Pediatric Clinical Effectiveness, Children’s Hospital of Philadelphia, Philadelphia, Pennsylvania, United States; d Division of Gastroenterology, Hepatology, and Nutrition, Children’s Hospital of Philadelphia, Philadelphia, Pennsylvania, United States; e Department of Pediatrics, University of California Davis, Sacramento, California, United States

**Keywords:** Preterm, microbiome, low-risk, sepsis, vertical transmission

## Abstract

Antibiotics are administered near-universally to very low birth weight (VLBW) infants after birth for suspected early-onset sepsis (EOS). We previously identified a phenotypic group of VLBW infants, referred to as low-risk for EOS (LRE), whose risk of EOS is low enough to avoid routine antibiotic initiation. In this cohort study, we compared 18 such infants with 30 infants categorized as non-LRE to determine if the lower risk of pathogen transmission at birth is accompanied by differences in microbiome acquisition and development. We did shotgun metagenomic sequencing of 361 fecal samples obtained serially. LRE infants had a higher human-to-bacterial DNA ratio than non-LRE infants in fecal samples on days 1–3 after birth, confirming lower bacterial acquisition among LRE infants. The microbial diversity and composition in samples from days 4–7 differed between the groups with a predominance of *Staphylococcus epidermidis* in LRE infants and Enterobacteriaceae sp. in non-LRE infants. Compositional differences were congruent with the distribution of virulence factors and antibiotic resistant genes. After the first week, the overall composition was similar, but changes in relative abundance for several taxa with increasing age differed between groups. Of the nine late-onset bacteremia episodes, eight occurred in non-LRE infants. Species isolated from the blood culture was detected in the pre-antibiotic fecal samples of the infant for all episodes, though these species were also found in infants without bacteremia. In conclusion, LRE infants present a distinct pattern of microbiome development that is aligned with their low risk for EOS. Further investigation to determine the impact of these differences on later outcomes such as late-onset bacteremia is warranted.

## Introduction

Very low birth weight infants (VLBW, birth weight <1,500 g) are at an elevated risk of early-onset sepsis (EOS, sepsis onset ≤3 days after birth), caused by ascending colonization and invasive infection of the newborn by microorganisms residing in the maternal genitorectal area.^1–4^ High prevalence of risk factors for EOS, and the overlap between signs of EOS and those arising from physiological instability inherent to prematurity, make identification of EOS immediately after birth challenging among preterm infants.^3–5^ As a result, approximately 80–90% of VLBW infants are adminitered empiric antibiotics after birth to ‘rule-out’ EOS, despite only 1–2% of all VLBW infants having confirmed infection.^5,6^ In work aimed to better target antibiotic administration for suspected EOS among very preterm infants, we identified a phenotype, named ‘low-risk for EOS’ (LRE), that had a risk of EOS low enough to be managed safely without routine antibiotic initiation.^5,7–9^ LRE phenotype is defined by the following criteria: (1) birth by cesarean delivery, (2) absence of spontaneous or induced labor, (3) rupture of membranes only at delivery, and (4) noninfectious medical indication for birth such as maternal preeclampsia or fetal growth restriction. While associated with reduced transmission of pathogens from mother to infant, the defining birth characteristics suggest that infants with the LRE phenotype may also differ in their acquisition of pioneer microbiome colonizers. The American Academy of Pediatrics (AAP) endorsed the identification of LRE infants as a risk-stratification approach for minimizing unnecessary antibiotic exposure.^10^ When managed per the AAP guidelines, LRE infants also differ from other preterm infants in the lack of exposure to systemic antibiotics immediately after birth,^8^ an exposure that is associated with microbiome changes and is a confounder in determining the natural history of the preterm microbiome.^11–17^

Understanding the dynamics of microbiome acquisition and development in LRE infants is specifically important in answering two clinical questions. First, is there molecular evidence of lower bacterial acquisition at birth that parallels the epidemiologically observed lower risk of EOS? Second, do the differences in birth environment and absence of routine antibiotic exposure after birth alter subsequent microbial ontogeny among preterm LRE infants compared to other preterm infants? To answer these questions, upon adopting the AAP delivery criteria-based approach for EOS risk assessment among the preterm infants,^8^ we enrolled a cohort of 48 VLBW infants and analyzed serial fecal samples as a marker of gut microbiome changes. We hypothesized that fecal samples obtained soon after birth from infants who met LRE criteria would have lower bacterial acquisition compared to infants born outside the pre-specified criteria (non-LRE). We also hypothesized that the microbiome composition would differ between the groups and that the difference would persist beyond the first three days after birth, a time period that is commonly used to demarcate differences in infection-causing colonization patterns among continuously hospitalized preterm infants.^3^ We assessed differences in virulence factors and antibiotic resistance genes in the two groups to further delineate differences arising from any compositional dissimilarities. Finally, we analyzed all study infants evaluated for late-onset bacteremia (onset >3 days after birth), to determine acute change in microbiome parameters with proven and suspected infection.

## Results

### Study population and clinical characteristics

**Figure 1.**
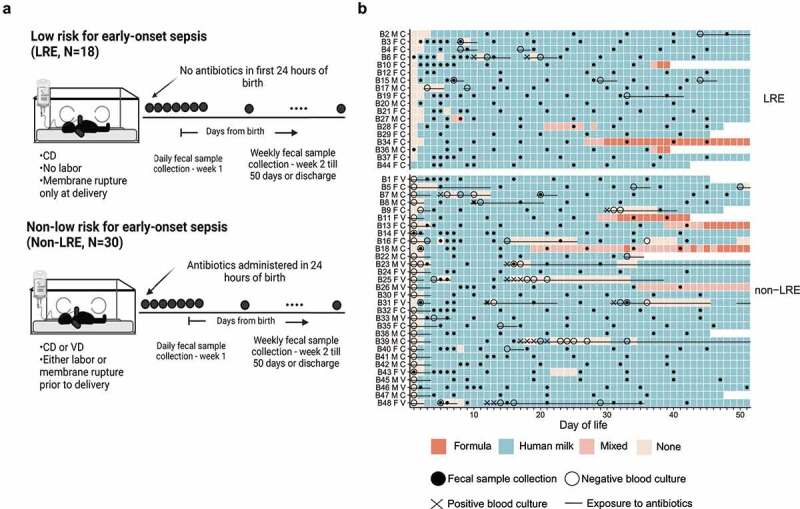
Clinical characteristics of the study cohort. (a) Depiction of study design and comparison groups. (b) Timeline of diet, clinical culture results, antibiotic exposure, and fecal sample collection. Each square of the grid represents one day in the life of an infant enrolled in the study. Infant study IDs are followed by codes to indicate sex (F = female; M = male) and delivery mode (C = cesarean delivery; V = vaginal delivery). A filled black dot (•) denotes a fecal sample, a large open circle (O)denotes a negative blood culture, a diagonal cross (✕) denotes a positive blood culture, and a line (―) denotes antibiotics.

**Abbreviations**: – CD, cesarean delivery; VD, vaginal delivery.

The study included 48 VLBW infants, 18 classified as LRE and 30 classified as non-LRE (Figure 1(a)). Table 1 highlights key differences and similarities in these groups. Maternal and infant characteristics were not different between the groups other than characteristics that define the LRE phenotype and factors that drive or are influenced by LRE defining criteria. These included, among LRE infants, higher incidence of maternal preeclampsia and fetal growth restriction, lower maternal antibiotic exposure during birth admission, lower neonatal antibiotic exposure (*P* < .001, Figure S1(a)) and a higher median gestational age (30 vs 28 *P* = .01) at birth. Aligned with current clinical practice, all non-LRE infants underwent an EOS evaluation and received antibiotics in the first 24 hours after birth while none of the LRE infants underwent such evaluation or received antibiotics. None of the cultures obtained for suspected EOS were positive. After 24 hours, both groups were exposed to different types of antibiotics (Figure S1(b)) and the proportion of LRE infants (39%) and non-LRE infants (43%) started on antibiotics was not different (*P* = .77). There were 10 episodes of late-onset bacteremia, 1 in a LRE infant, and 9 episodes among 8 non-LRE infants (Figure 1(b)).

**Table 1.** Population characteristics.

Characteristics	LRE	Non-LRE	*P* value
**Maternal characteristics**	**N = 16**	**N = 28**	
Age, median (Q1, Q3)	32 (27, 35)	32 (28, 34)	0.86
Race, n (%)			0.31
Black	6 (38)	13 (46)	
White	7 (44)	13 (46)	
Asian	1 (6)	2 (7)	
Other/unknown	2 (13)	0	
Hispanic ethnicity^1^, n (%)	1 (6)	3 (11)	0.54
Gravidity, n (%)			0.16
1	9 (56)	8 (29)	
2	4 (25)	8 (29)	
≥3	3 (19)	12 (43)	
Multiple gestation pregnancy, n (%)	3 (19)	6 (21)	>0.99
Antenatal steroids, n (%)	16 (100)	27 (96)	>0.99
Antenatal antibiotics during delivery admission (excluding surgical skin prophylaxis)^2^, n (%)	2 (12.3)	24 (85.7)	<0.001
Group B *Streptococcus* colonization status, n (%)			0.91
Positive	4 (25)	7 (25)	
Negative	8 (50)	16 (57)	
Unknown	4 (25)	5 (18)	
Maternal co-morbidity, n (%)^3^			
Preeclampsia	13 (81.3)	3 (10.7)	<0.001
Fetal growth restriction	14 (87.5)	3 (10.7)	<0.001
Prelabor premature rupture of membrane	0	10 (37.7)	<0.001
Spontaneous onset of preterm labor	0	24 (85.7)	<0.001
Abruption	0	3 (10.7)	<0.001
Diagnosis of clinical chorioamnionitis	0	1 (4)	>0.99
LRE defining characteristics			
Cesarean delivery, n (%)	16 (100)	14 (50)	<0.001
ROM prior to delivery, n (%)	0	22 (79)	<0.001
Duration of membrane rupture (in hours), median (Q1, Q3)	0 (0, 0)	3 (0, 32)	<0.001
Labor onset prior to delivery, n (%)	0	23 (82)	<0.001
**Infant characteristics**	**N = 18**	**N = 30**	
Gestational age, median (Q1, Q3)	30 (28, 31)	28 (26, 29)	0.01
Birth weight, median (Q1, Q3)	1080 (745, 1285)	1095 (825, 1295)	0.64
Sex (female), n (%)	12 (67)	16 (53)	0.55
Diet			
First day of enteral feed, median (Q1, Q3)	1 (1, 2)	2 (1, 2)	0.58
Percent days with no enteral feeds (of total days in the study), median (Q1, Q3)	3 (2, 6)	4 (2, 12)	0.12
Percent days with human milk-based diet (of total days in the study), n (%)^4^			0.64
≤50%	0	0	
>50% to 75%	1 (6)	4 (13)	
>75% to 100%	17 (94)	26 (87)	
Culture confirmed systemic infection^5^			
BSI/meningitis ≤3 days after birth, n (%)	0	0	N/A
BSI/meningitis >3 days after birth, n (%)	1	8 (27)	0.13
Antibiotic exposure			
Infants started on antibiotics ≤24 hours after birth, n (%)	0	30 (100.0)	<0.001
Infants started on antibiotics >24 hours after birth, n (%)	7 (39)	13 (43)	>0.99
Fecal samples analyzed per infant, median (Q1, Q3)	8 (7, 9)	7 (6, 8)	0.12
Disposition at 50 days, n (%)			0.83
In hospital	13 (72)	23 (77)	
Discharged home or transferred	5 (28)	6 (20)	
Deceased	0	1 (3)	

**Notes**: ^1^Ethnicity missing for 1 mother of LRE infant. ^2^Surgical skin prophylaxis defined as (a) cefazolin, or (b) clindamycin and gentamicin, as the only antibiotics administered during delivery admission, within 60 minutes of delivery. ^3^Mothers may have more than one co-morbidity; fetal growth restriction for any fetus in multiple gestation births is included. ^4^Human milk-based diet includes a base diet of mother’s milk or donor human milk. ^5^Bloodstream infection was defined as isolation of a pathogen from blood culture. Coagulase-negative staphylococci were not considered a pathogen when isolated ≤3 days after birth. After 3 days from birth, coagulase-negative staphylococci were considered a pathogen if the clinical team treated the infant with appropriate antibiotics for ≥5 days.

**Abbreviations**: – BSI, blood-stream infection; LRE, low-risk for early-onset sepsis; ROM, rupture of membranes; N/A, not applicable.

Dietary regimens at our center are agnostic to delivery criteria and were similar between the groups. Both groups predominantly received human milk (mother’s own milk or pasteurized donor human milk) as the base diet on 90% of days in the study. Infants received formula on 5% of days in the study; 55% of formula was in combination with human milk. The consumption of human milk and formula was not different between the groups (*P* = .1 and 0.7, respectively; Figure S1(c)). Milk from human donors represented 17% of days when human milk was consumed. The amount of donor milk consumption decreased with age (*P* < .001) and was not different between groups (*P* = .7, Figure S1(c)). Infants had no dietary intake on 7.4% of days in the study, and a majority of such days were within the first two weeks of life (Figure 1(b)). In the first two weeks of life, the number of days without enteral feeds was not different between the infant groups (*P* = .5). After two weeks of life, non-LRE infants had a greater number of days without enteral feeds, compared to the LRE infants (*P* = .003, Figure S1(d)), often in association with periods of bacterial infection (Figure 1(b)). Fortification increased with age (Figure S1(e)) in both groups with more non-LRE infants receiving human milk-based fortifier, likely reflecting the difference in gestational age distribution and the unit’s policy to offer human milk-based fortifier only to infants of <1,000 g birth weight. Thus, despite the differences between the groups associated with delivery criteria, other demographics, and diet were comparable between the groups.

A total of 361 fecal samples were collected from infants during the first 50 days after birth, with a median of 7 samples per infant (IQR 7–9). The fecal samples were submitted for shotgun metagenomic sequencing to measure the microbiome, yielding a median of 2.8 M paired-end reads per sample after quality control (IQR 2.2–4.5). The sequencing output for one sample did not meet our minimum quality thresholds and was excluded from further analysis.

### Fecal microbiome differs between LRE and non-LRE infants during the first week of life

**Figure 2.**
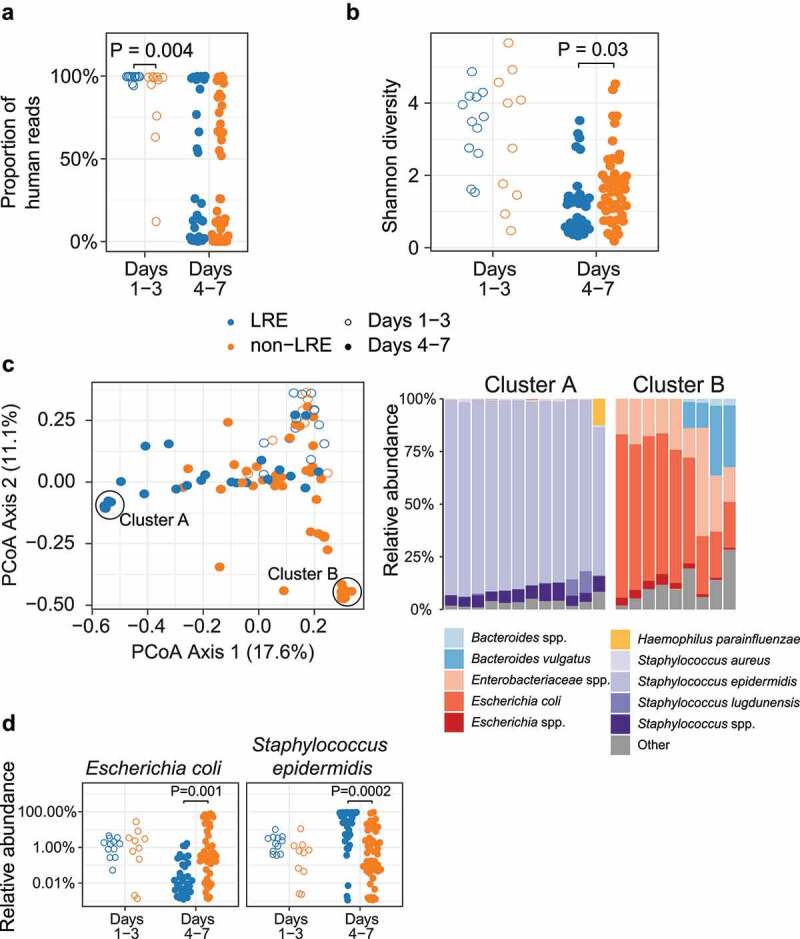
Microbiome of LRE and non-LRE infants in the first week of life. Fecal samples of infants are shown as open circles when obtained on day 1–3 (0–72 h) after birth and closed circles when obtained on day 4–7 (72–168 h) after birth. (a) The proportion of human reads obtained in shotgun metagenomic sequencing was higher in LRE infants on days 1–3 but not different on days 4–7. (b) The Shannon diversity of bacterial species was not different on days 1–3 but was lower in LRE infants on days 4–7. (c) Comparison of bacterial community composition based on Bray-Curtis distance between samples, and taxon relative abundance in two high-density sample clusters. (d) Relative abundance of taxa (logarithmic scale) found to be different between LRE and non-LRE infants on days 4–7.

**Abbreviation**: – LRE, low-risk for early-onset sepsis.

We first examined shotgun metagenomic sequencing samples obtained during the first week of life to compare the amount and characteristics of the microbiome during this distinctive early window. Shotgun metagenomic sequencing yields sequence reads from microbial populations and from the host when host DNA is present. When the fecal microbial density is low, as in fecal samples obtained shortly after birth, the percentage of host-derived sequence reads can increase to nearly 100%.^18^ Thus, a high percentage of host-annotated reads in shotgun metagenomic sequencing is a marker of low microbial density, manifested as a low microbial-to-host DNA ratio.^18^ Both groups exhibited a high percentage of host DNA, suggesting an overall low bacterial content after birth. The fraction of host reads was higher in LRE infants during the first three days after birth compared to non-LRE infants (*P* = .004, Figure 2(a)). On days 4–7 of life, the percentage of host DNA decreased in both groups, and the level of host DNA was not different between infant groups (*P* = .13). The direction of the difference in day 1–3 is consistent with the hypothesis that LRE and non-LRE infants differ in bacterial acquisition at birth and that increased exposure to maternal bacteria among non-LRE infants leads to a more rapid initial increase in fecal bacteria compared to LRE infants.

Next, we compared microbiome diversity and composition and found several differences between the groups in days 4–7 of life. Microbial alpha diversity was not different between infant groups during the first three days after birth but was higher in the non-LRE group on days 4–7 (*P* = .03, Figure 2(b)). The general composition of the bacterial community, determined by Bray-Curtis distance (a measure of beta diversity), was not different between the groups during the first three days after birth (*P* = .1) but differed on days 4–7 (R^2^ = 0.08, *P* = .001, Figure S2). In the ordination of Bray-Curtis distance, we noticed two high-density sample clusters at opposite ends of the first principal coordinate axis (Figure 2(c)). Samples in high-density cluster A, arising predominantly from the LRE group (10 of 12 samples), contained a high relative abundance of *Staphylococcus epidermidis*. Samples in high-density cluster B, arising exclusively from the non-LRE group, contained a high relative abundance of *Escherichia coli* and other *Enterobacteriaceae* species (Figure 2(c)). We compared the relative abundance of bacterial species and found differences between the groups on days 4–7 that mirrored our observation in the high-density clusters. On days 4–7 of life, the relative abundance of *S. epidermidis* was higher in infants in the LRE group (*P* = .0002), while the relative abundance of *E. coli* was higher in infants in the non-LRE group (*P* = .001, Figure 2(d)). Thus, we found that the LRE phenotype differed in microbiome composition during days 4–7 of life, particularly with regard to the primary colonizers *S. epidermidis* and *E. coli*.

### Differences in the fecal microbiome of LRE and non-LRE infants after the first week of life

**Figure 3.**
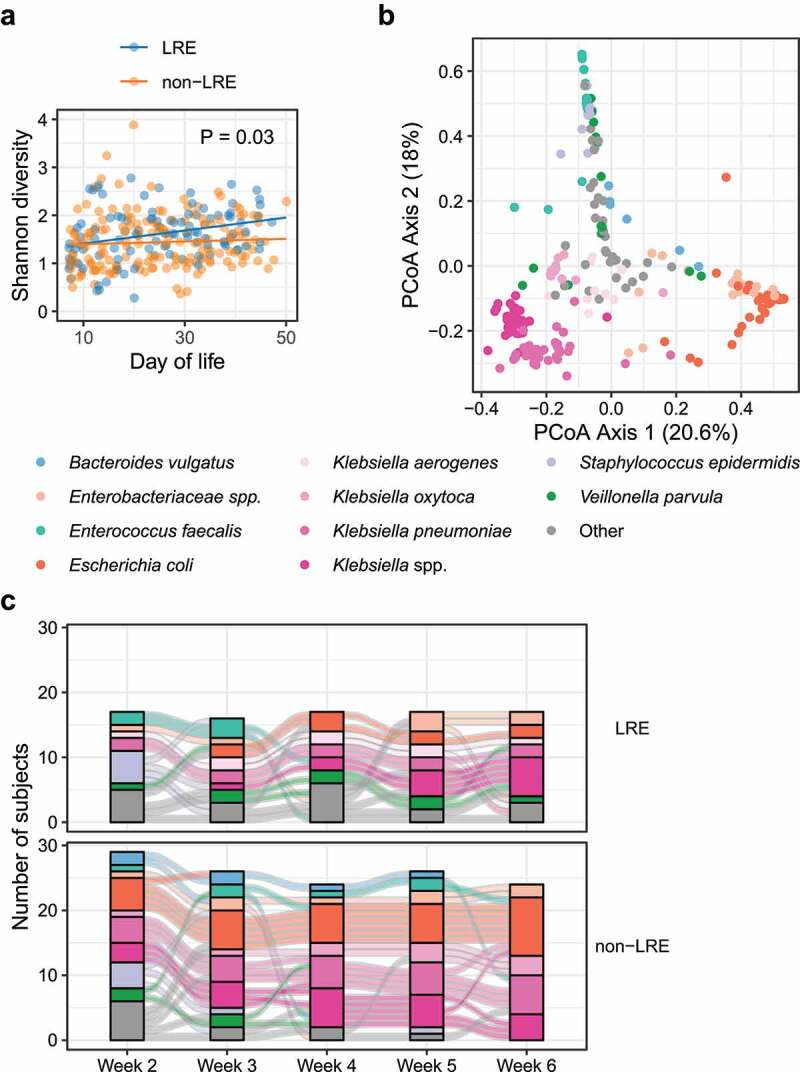
Microbiome of LRE and non-LRE infants after the first week of life. (a) Shannon diversity of samples after the first week of life. (b) Comparison of bacterial community composition by Bray-Curtis distance between samples. The species with highest abundance in each sample is indicated by color. (c) Time course of the most abundant species in infant fecal samples after the first week of life. Stacked bars at each time point indicate the most abundant species in each sample. Curves connect samples from the same infant that were collected at consecutive time points.

**Abbreviation**: – LRE, low-risk for early-onset sepsis.

We next assessed for differences in microbiome diversity and composition after the first week of life to determine if the difference noted in days 4–7 endured. After the first week of life, microbial alpha diversity increased in the LRE group (*P* = .0002) but not in the non-LRE group (*P* = .4). Consequently, microbial diversity was different between the two groups during this time (*P* = .03, Figure 3(a)). However, bacterial community composition was not different between groups (*P* = .3, Figure S3(a)). While day of life was associated with bacterial community similarity (R^2^ = 0.05, *P* = .001), and despite the rapid development of the fecal microbiome during this period, subject identity had the largest association with bacterial community similarity (R^2^ = 0.6, *P* = .001). This suggests substantial inter-subject variability that is not explained by other factors.

Bacterial communities did not form dense clusters, as in samples collected during the first week of life but were instead arranged along gradients corresponding to the first and second axes based on Principal Coordinates Analysis (Figure 3(b)). The Axis 1 gradient extended from samples where the most abundant taxon was *Klebsiella* spp. to samples dominated by *E. coli* and unclassified *Enterobacteriaceae*, while the gradient along Axis 2 extended from the *Klebsiella*/*E. coli* samples toward samples with *S. epidermidis* and *Enterococcus faecalis* as the most abundant species.

In both LRE and non-LRE infants, the number of samples dominated by *S. epidermidis* decreased with the age of the infant, and the number of samples dominated by *Enterobacteriaceae* (including *Klebsiella* spp. and *E. coli*) increased (Figure 3(c)) with age. While the most abundant taxon was not different between LRE and non-LRE infants, the relative abundance of several taxa differed with increasing age: *E. coli* (*P* = .02), *Enterobacter cloacae* (*P* = .0003), and *Citrobacter freundii* (*P* = .002) increased more in LRE infants, whereas *E. faecalis* (*P* = .03) increased to a greater degree in non-LRE infants (Figure S3(b)). Our models also indicated that the relative abundance of *E. coli* was initially lower in the LRE infants (*P* = .04) and that the abundance of *E. faecalis* was initially lower in non-LRE infants (*P* = .04). In contrast, other taxa that changed with age did not differ between LRE and non-LRE infants: *S. epidermidis* decreased, and *Klebsiella* spp, *E. coli, C. freundii, Veillonella parvula*, and *E. cloacae* increased (Figure S3(c)) with age in both groups. The differences in the trajectory of these taxa between LRE and non-LRE infants suggest differential species-specific risk for late-onset bacteremia in the two groups.

### Differences in virulence factor and antibiotic resistance gene abundance

Having characterized taxonomic differences between LRE and non-LRE infant microbiomes, we conducted a functional analysis of bacterial genes. Here, we focused on the abundance of virulence factors and antibiotic resistance genes that would be relevant to sepsis risk and severity.

**Figure 4.**
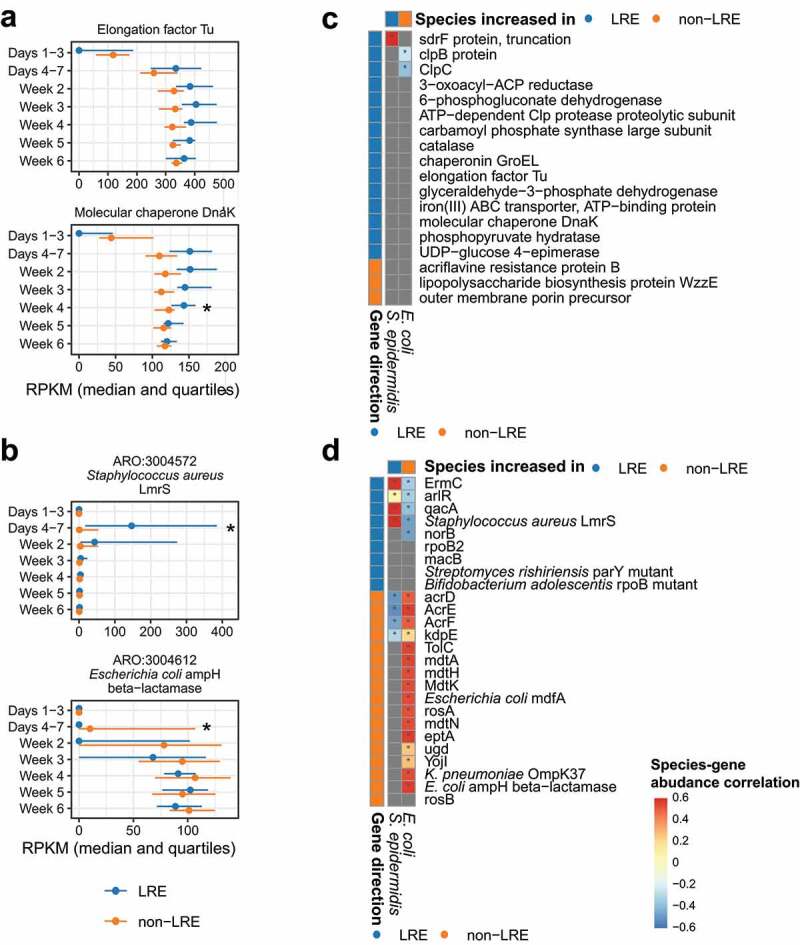
Virulence factors and antibiotic resistance genes in LRE and non-LRE infants. (a) Selected virulence factors and (b) antibiotic resistance genes found to be different between LRE and non-LRE infants (complete set in Figure S4 and Figure S5, respectively). In each plot, the x-axis has units of reads per kilobase gene length per million reads (RPKM). Lines extend from the first to the third quartile, and the median is indicated by a point. An asterisk indicates a difference in gene abundance between LRE and non-LRE infants after controlling for a 5% false discovery rate. (c) Partial correlation analysis of virulence factor and (d) antibiotic resistance gene abundance and species abundance. Grey squares represent genes not found in the species based on a database search. An asterisk indicates a statistically significant correlation after controlling for a 5% false discovery rate.

**Abbreviation**: – LRE, low-risk for early-onset sepsis.

To examine if the groups differed in prevalence of virulence factor-related genes, we tabulated the abundance of virulence genes from the Virulence Factors Database (VFDB) in each sample and compared the samples from the two study groups during successive time periods. Notably, we observed three genes that were more abundant among non-LRE infants, all of them differing only on days 4–7 (Figure S4). The three genes, *acrB, WzzE* (lipopolysaccharide biosynthesis protein), and outer membrane porin precursor, encoded structural or membrane proteins. These differences mainly tracked to the same time period where differences in microbiome composition were noted (days 4–7 of life) and the genes identified were largely absent in LRE infants. Also consistent with the dissipation of differences in microbiome composition after one week, these genes became more prevalent over time, and the abundance was not different between the study groups after day 7. We also identified 15 genes that were increased in LRE infants, including genes annotated as *dnaK* and elongation factor Tu (Figure 4(a) and Figure S4). The relative abundance among the 15 genes increased during the first 4 weeks after birth but we did not detect differences in the abundance of the genes after week 4. Although variants of such genes are implicated in virulence for some species, the 15 genes identified were concentrated in housekeeping and core metabolism functions.

In analyzing antibiotic resistant genes (ARG), we focused on genes conferring resistance to methicillin, gentamicin, and ampicillin, due to the clinical importance of these antibiotics in neonatal care. We selected 15 such ARGs from the Comprehensive Antibiotic Resistance Database (CARD) and compared their relative abundance between groups. The abundance of one gene was found to be different: *mecA*, conferring resistance to methicillin, was higher in LRE infants on days 4–7, where it reached a median of 50 reads per kilobase per million (RPKM) (Figure S5). This difference correlates with the observed increase in the relative abundance of *S. epidermidis* in the LRE group during this time period (Figure 2(d)). By day 15, the *mecA* gene was absent in both groups for the remainder of the study. Although all non-LRE infants received ampicillin and gentamicin during the first 24 hours after birth, we did not observe an increased abundance of genes associated with resistance to these antibiotics during any time period.

We also performed an untargeted comparison of the 50 most abundant ARGs from the CARD. Here, we identified nine genes that were increased in LRE infants and 17 genes that were increased in non-LRE infants (Figure 4(b), Figure S5). Mirroring our analysis of virulence factors, the differences in non-LRE associated genes occurred exclusively on days 4–7, where the gene was acquired slightly earlier in the course of time for these infants. Likewise, the increase in LRE associated genes was limited to the first 4 weeks of life and included genes for housekeeping and core metabolism.

Because we observed differences in the virulence factors and ARGs that mirrored the taxonomic differences observed on days 4–7, we sought to determine whether the gene abundances had a statistically significant correlation with the two species we found to be different during that time period: *E. coli* and *S. epidermidis*. For example, we wished to know whether the *E. coli* ampH beta−lactamase gene abundance specifically correlated with *E. coli* abundance in the non-LRE group more than would be expected after accounting for the clinical group, mode of birth, and day of life. To answer this question, we first verified by database search that the gene had been previously observed in genomes from *E. coli* or *S. epidermidis*. For genes that were plausibly present in both the species, we then computed the residual abundances of the gene and species after factoring out group differences, and then tested for a gene-species correlation. For the virulence factors, we observed that only a small number were present in *E. coli* and *S. epidermidis* genomes (Figure 4(c)), indicating that the species were unlikely to account for the observed differences in virulence factor abundances. However, we observed strong positive correlations between *E. coli*/*S. epidermidis* abundance and the ARG abundances (Figure 4(d)), suggesting that the two species may play host to the temporary differences in ARG reservoir observed in the range of days 4–7 after birth.

### Bacterial infections and microbiome alterations

**Figure 5.**
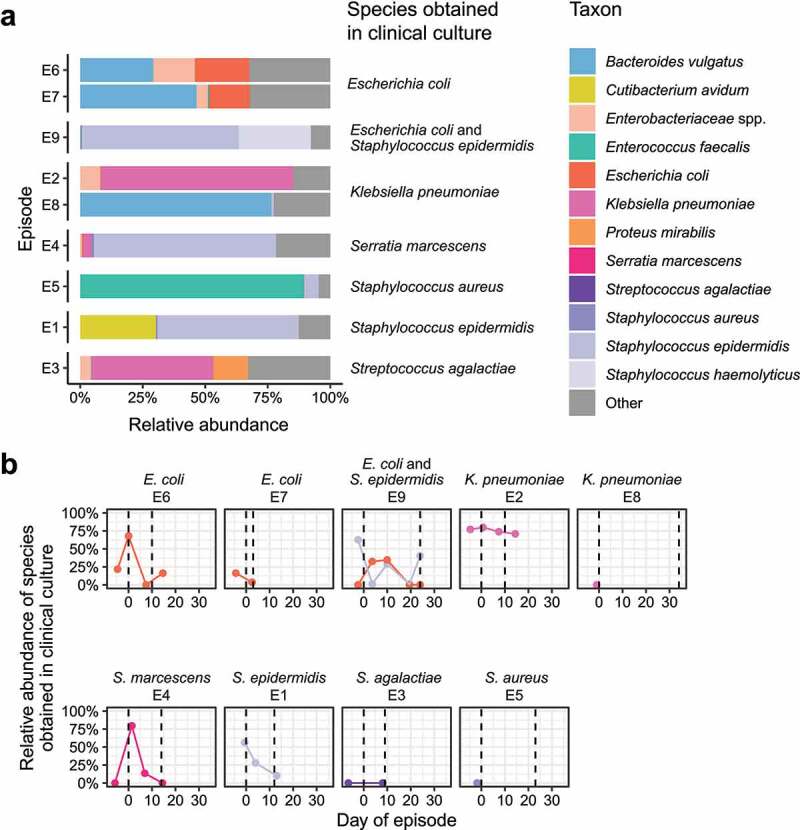
Fecal microbiome during episodes of bacteremia. (a) Relative abundance of bacterial species in pre-antibiotic fecal samples obtained prior to clinical evaluation resulting in the diagnosis of bacteremia. (b) Time course of relative abundance for the bacterial species matching those obtained from blood culture in each episode. Dashed lines indicate the days that antibiotics started and ended during each episode. Episodes E5 and E8 did not have post-antibiotic samples. The final sample in episode E7 was obtained prior to completion of therapy, as the infant was transferred to a different facility.

**Abbreviation**: – LRE, low-risk for early-onset sepsis.

To understand colonization patterns associated with bacteremia in the two groups, we analyzed fecal samples around late-onset bacteremia episodes, defined as isolation of a pathogen >3 days after birth in the cohort. The study infants had 10 such episodes and pre-antibiotic fecal samples were available for 9 episodes occurring in 8 infants (Table S1, Figure S6(a)). Pre-antibiotic fecal samples were collected for a median of 4 days (IQR 3–5) before the blood sample that isolated the cultured organism. We analyzed the pre-antibiotic fecal samples for the presence and relative abundance of the species obtained clinically from blood culture. The species cultured from blood was identified in the pre-antibiotic fecal sample in all bacteremia episodes, though relative abundance in feces was not consistently high for any given species (Figure 5(a), Figure S6(b)).

We next analyzed pre-antibiotic fecal samples from seven infants with eight episodes of infection evaluation where blood cultures were negative, antibiotics were discontinued after 3 days, and the infants remained well. We compared these with the pre-antibiotic fecal samples from the blood culture-positive episodes, (Table S1, Figure S6(c)). There was no difference in bacterial species richness (*P* = .4) or Shannon diversity (*P* = .3) at the pre-antibiotics time point between infants with and without bacteremia (Figure S6(d)). Moreover, the species identified in episodes of bacteremia – *E. coli, S. epidermidis, Staphylococcus aureus*, Group B *Streptococcus, Klebsiella pneumoniae*, and *Serratia marcescens* – were also present in pre-antibiotic fecal samples from infants who ultimately had negative blood cultures (Figure S6(e)).

We then examined the time course during seven of the blood culture-positive episodes where we had both pre- and post-antibiotic fecal samples, to assess the degree to which antibiotic exposure was associated with alterations in fecal microbiome composition over time. Antibiotic intervention did not eliminate the pathogen cultured in blood from the fecal bacterial community; in five (E1, E2, E4, E6, and E9) of seven episodes, the cultured species persisted in high relative abundance (Figure 5(b)). In episode E1, *S. epidermidis* remained at over 10% relative abundance throughout the time series. In episode E2, *K. pneumoniae* remained in high relative abundance in the feces before, during, and after antibiotic exposure. Likewise, after *S. epidermidis* was identified in blood during episode E9, it remained a prominent constituent of the microbiota despite fluctuations in species representation across the time series. In summary, bacterial species identified by blood culture were also identified in the feces of VLBW infants during episodes of bacteremia, though the relative abundance of the fecal species before and after antibiotic treatment did not follow a consistent pattern.

## Discussion

In this cohort study, we found that LRE infants demonstrated a distinct pattern of fecal microbiome acquisition and longitudinal change. Differences were most prominent in days 4–7 after birth with significant differences in microbial diversity, overall composition, and a predominance of *Staphylococcus* sp. colonization among LRE infants compared to non-LRE infants. These compositional changes were accompanied by congruent differences in virulence factors and ARGs. After the first week, we noted differences in diversity, but not overall composition. However, the change with age in the relative abundance of several taxa, many of which are important late-onset sepsis (LOS)-causing pathogens, remained different between groups. Infants with the LRE phenotype constitute a third of very preterm infants.^5,8,9^ Thus, differences in microbiome development in this group can impact our understanding of the microbiome in preterm infants as a population and inform the design of interventions that manipulate the microbiome to improve outcomes, such as decolonization measures and probiotic use.

LRE infants present with a combination of clinical factors that suggest a low risk of bacterial transmission from mother to baby, each of which also has an independent association with microbiome development.^19,20^ We chose to study LRE infants as a phenotype rather than identify the contribution of each delivery criteria to the microbiome separately because these factors do not occur independently in practice. The decision for cesarean delivery without trial of labor clusters with specific indications for preterm delivery, such as maternal preeclampsia and fetal growth restriction; these factors influences maternal indications for antibiotic administration; and are potentially associated with other unmeasured differences of maternal and neonatal host immune state at birth (Table 1). ^21,22^ Together these create a profile that is best captured as a ‘phenotype’ especially relevant to designing clinical interventions.^20^ The restriction of antibiotic initiation in LRE infants is an illustrative example for such an intervention and has additional implications for the developing neonatal microbiome. Traditionally, antibiotics were administered to 80–90% of VLBW infants just after birth.^5,7^ Studies that inform our current understanding of microbiome development in very preterm infants often accept this near collinearity as an inevitable part of care required by preterm infants.^11,12,14–16^ Widespread use of the AAP guidelines to limit antibiotic initiation in LRE infants could change antibiotic exposure and alter early microbiome development by limiting the effects of early antibiotics.^8,9,13^ However, the limiting of antibiotic initiation is based on the combination of delivery criteria rather than any one delivery crtiera in isolation; therefore studying the group as a phenotype is clinically relevant.

The pathogenesis of EOS is based on mother to child microbial transmission.^23^ While epidemiological evidence for rarity of EOS in LRE infants is reported,^5,7–9^ evidence of lower microbial acquisition can further inform risk estimation for EOS in these infants. Using the human-to-bacterial DNA ratio in meconium samples, we deduced that LRE infants had less bacterial DNA than non-LRE infants in days 1–3 after birth. This finding extends the clinical observation of lower acquisition of pathogens, to the lower acquisition of all colonizers for LRE infants. Our findings are reassuring for clinicians who remain concerned about managing LRE infants without antibiotic initiation after birth and support the EOS management approach recommended by AAP.^10^

Many studies describing the preterm microbiome development are influenced by high incidence of antibiotic administration to very preterm infants just after birth.^11–17^ We had the opportunity to study LRE infants who avoided this exposure. Preterm microbiome development is described to occur in stages with early colonization by Firmicutes followed by an increase in Gammaproteobacteria (this class includes the family Enterobacteriaceae), and lastly an increase in anaerobes such as Clostridia and *Bacteroides*.^14,24^ We saw a similar pattern with decrease in *Staphylococcus* sp. and an increase in Enterobacteriaceae sp. with age in the overall cohort. However, there were some notable differences between LRE and non-LRE infants in the first week of life. The differences align with the findings of Greenwood et al.,^13^ who compared the fecal microbiome of 13 very preterm infants without antibiotic exposure with 48 infants exposed to 1–4 days of antibiotics in the first week. The former group met many of the criteria to be classified as LRE infants and, similar to our findings, had *Staphylococcus* sp. as the most abundant genera. In contrast, Enterobacteriaceae was the most abundant taxa in infants exposed to early antibiotics. Studies also report *Staphylococcus* sp. and other Firmicutes as frequent early colonizers among infants born by cesarean delivery (all LRE infants) compared to the higher abundance of intestinal colonizers such as *E. coli* in infants born vaginally (half of non-LRE infants).^20,25,26^ Differences in virulence factors were more frequent on the days 4–7, aligned with differences in diversity and composition during this time. Differences in ARGs also followed the pattern of microbiome differences observed on days 4–7, with ARGs observed in LRE infants commonly found in *Staphylococcus* sp. and those observed in non-LRE infants commonly found in Enterobacteriaceae.^27–29^ The higher *mecA* among LRE infants, a gene conferring resistance to *S. aureus* against methicillin, potentially reflects the abundance of *S. epidermidis*, a known reservoir of *mecA*;^30^ none of the infants were directly exposed to the methicillin group of antibiotics. Others have noted this phenomenon of ARG acquisition in neonates without direct exposure to antibiotics.^12^ It is also notable that similar to other studies, short exposure to ampicillin and gentamicin in the non-LRE group was not associated with increased isolation of ARG for these antibiotics.^43^

LRE infants who started with a lower proportion of *Enterobacteriaceae* saw greater gains in this taxon, while non-LRE infants saw a greater rise in *E. faecalis*. Colonization patterns can drive the risk of invasive infection for some pathogens.^24,31–33^ Epidemiological studies have attributed differences in the frequency of LOS to maternal hypertension and fetal growth restriction – both prominently associated with LRE infants.^34,35^ While the current study is not powered to detect such associations, our findings suggest that delivery criteria, as represented in LRE infants, could modify early antibiotic exposure and be a modifying effect for species-specific colonization pressures at different periods from birth.

Multiple studies have shown that the fecal microbiome is a viable reservoir and source of origin for LOS pathogens.^36–40^ The species ultimately cultured in clinical specimens is frequently identified in fecal samples obtained 1–3 days before the sentinel event.^37,40^ We were able to detect the pathogen species causing bacteremia in pre-antibiotic fecal samples, but unlike some studies,^40^ we did not find a consistently high abundance in all samples. We also detected the same species in fecal samples of infants without bacteremia and found no difference in the diversity of the microbiome in pre-antibiotic fecal samples obtained from infants with and without bacteremia. Finally, we found that the species causing bacteremia remained detectable in fecal samples, sometimes without a change in abundance, despite multiple days of appropriate intravenous antibiotic treatment and clearance of bacteremia. One possibility for the differences in our findings compared to others is that 5 of the 9 bacteremia episodes were central line-associated bloodstream infections (CLABSI), that have been excluded by others when considering the fecal microbiome as a source for bacteremia.^36^ However, there is likely an overlap between CLABSI and gut microbe-associated LOS.^41^ Overall, our findings suggest that in addition to the presence of the pathogen in the gut microbiome, other (potentially host-related) factors are critical in driving invasive infection.^38^ Our findings also highlight the difficulty in attaining true decolonization and possible differences in the magnitude of the effect of antibiotics on the gut microbiome when administered intravenously compared to the well-known impact of oral administration.^17,42–44^

The strengths of our study include the prospective design, detailed phenotyping of infants, and use of deep sequencing techniques. Our study has limitations. Studies describing the preterm microbiome have highlighted the importance of corrected gestational age over chronological age.^14^ We chose to present the data by chronological age as our goal was to align with the clinical definitions for changing risk of infection type (early- versus late-onset sepsis) that currently follows chronological age. Due to the limited number of LOS events in our study, our analysis of LOS events was exploratory, representing an avenue of research we intend to pursue in future studies. We see our results as hypothesis generating. The small sample size of the study, while similar to other metagenomics studies,^36,40^ along with the smaller number of samples comparing early microbiome differences suggests the need for validation in larger populations.

## Conclusion

We found that infants clinically categorized as low risk for EOS also had differences in microbiome acquisition and development, most prominently in the first week of life, as compared to non-LRE infants. Our findings warrant further studies to understand the long-term outcomes among infants with this clinically and microbiologically unique phenotype.

## Patients and methods

### Study setting and design

This was a prospective cohort study of infants admitted to Pennsylvania Hospital neonatal intensive care unit born with birth weight <1,500 g and gestational age <33 weeks. Mothers of all subjects consented to participation, and the study was approved by the University of Pennsylvania Institutional Review Board. The study period included admissions between March 2018 and October 2019. A total of 75 infants were enrolled during this study period: 29 LRE and 46 non-LRE infants. The 48 infants (18 LRE and 30 non-LRE) included in the current study were chosen as a pragmatic sample that represented the distribution of delivery criteria and includes all cases of late-onset bacteremia. Probiotics are not routinely prescribed at the study site.

### Sample collection

Samples were collected daily, as available, in week 1 after birth, and weekly thereafter. Samples were stored at 4°C for <48 hours prior to being frozen in a − 80°C freezer. Median duration between collection and freezing was 6.4 hours (IQR 3.0–13.6). In total, sequences from 361 fecal samples obtained from 48 infants from birth to 50 days of age, were included in this study. Age at which sample was collected was calculated in hours by differencing the date and time of birth from the date and time of collection. Distribution of samples over time per patient is depicted in Figure 1(a). Table S2 shows the distribution of patients and samples per time period.

### Data collection and study definitions

Clinical data including demographics, maternal information, daily diet details, clinical care details and clinical outcomes at the time of final status were prospectively collected from electronic medical records and managed using REDCap electronic data capture tools hosted at the University of Pennsylvania.^45,46^ Maternal race, education, and occupation were collected in a questionnaire and self-reported by the mother. Late-onset bacteremia was defined as the isolation of a pathogen from blood after 3 days from birth. Aligned with other neonatal sepsis studies, coagulase-negative staphylococci were considered a pathogen if the clinical team treated the infant with appropriate antibiotics based on isolate susceptibilities for ≥5 days.^47^

### Analytic groups

Infants were categorized based on delivery criteria. Infants meeting all of the following were included in the LRE group: delivery by cesarean section, absence of labor, and rupture of membrane at the time of delivery. All other infants were included in the non-LRE group.

### Shotgun metagenomic sequencing

The Qiagen DNeasy PowerSoil kit was used for DNA extraction from infant fecal samples. Extracted DNA was quantified with the Quant-iT PicoGreen Assay Kit (Thermo Fisher). The NexteraXT kit was used to generate DNA libraries for shotgun metagenomic sequencing. Extraction blanks and DNA-free water samples were prepared for sequencing as negative controls. A laboratory-generated DNA mock community of *Vibrio campbellii* and Lambda phage was included as a positive control. Sequencing was performed on the Illumina HiSeq 2500 instrument, generating paired-end 125 bp reads.

### Bioinformatics analysis

Shotgun metagenomic data was processed using the Sunbeam bioinformatics pipeline using default parameters.^48^ Within Sunbeam, paired-end reads were trimmed for quality with Trimmomatic,^49^ low-complexity sequences were removed with Komplexity,^48^ and human reads were identified by alignment to the human genome with Burrows-Wheeler Alignment tool.^50^ Taxonomic assignments were generated with Kraken. Alignment to gene orthologs in the Virulence Factor Database and the Antibiotic Resistance Gene Database was carried out using Burrows-Wheeler Alignment tool.^50–52^

### Statistical analysis

To compare clinical characteristics between infants in the two categories, we used Fisher’s exact test or Mann-Whitney *U* test as appropriate. We included four pairs of twins and did not attempt to adjust for lack of independence. Mann-Whitney *U* test was used for comparison of human DNA levels and alpha diversity in microbiome samples within each time window during the first week of life. Differences in bacterial composition between groups were assessed using the PERMANOVA test on Bray-Curtis distance between samples.^53^ For the PERMANOVA test, random permutations were restricted to exchange infants between study groups or to exchange time point labels only within infants. Bacterial relative abundances were log-transformed and analyzed using linear mixed effects models with random intercepts for each subject. Gene abundances were analyzed separately at each time window using linear models.

For analysis of species correlation with virulence factor and antibiotic resistance genes, we first confirmed the presence of each gene in the species of interest, then carried out a partial correlation analysis to evaluate the gene-species correlation. The presence of genes was determined through a manual search on the CARD (https://card.mcmaster.ca/home) or VFDB (http://www.mgc.ac.cn/VFs/main.htm) website. The partial correlation analysis was undertaken by first constructing two linear mixed-effects models. The predictive variables for both models were birth category, day-of-life, the category-day-of-life interaction. The subject identifier of the infant was included as a random intercept to account for repeated measurements. The outcome variable was the CARD or VF gene for one model (log RPKM) and the species (log relative abundance) for the other model. Then, the residuals of the fixed effects from each of these models were correlated to each other for each gene/species pair using Pearson’s method.

When multiple comparisons were carried out, p-values from the tests were corrected to control for a 5% false discovery rate, using the method of Benjamini and Hochberg.^54^

## Supplementary Material

Supplemental Material

## Data Availability

The shotgun metagenomic sequencing data supporting the conclusions of this article is deposited at the NCBI Sequence Read Archive (PRJNA872399) and can be accessed at the URL https://www.ncbi.nlm.nih.gov/bioproject/PRJNA872399.
