# Supplementary material for: Preterm infants at low risk for early-onset sepsis differ in early fecal microbiome assembly
Source: Gut Microbes. 2022 Dec 6;14(1):2154091. doi: 10.1080/19490976.2022.2154091 (PMC9733690; doi:10.1080/19490976.2022.2154091)
Supplement: Supplemental Material [file KGMI_A_2154091_SM3804.pdf]

Table S1. Clinical details for episodes of bacteremia and negative infection evaluations

Episode #	Group	Blood culture DOL	Blood culture organism	Other culture obtained	Other culture organism	Indication for evaluation	Final Diagnosis[1]	Antibiotic type (days)[2]
**Episodes of bacteremia**								
1	LRE	9	*Staphylococcus epidermidis*	None	N/A	Increased apnea events and abdominal distension and discoloration	CLABSI	Gentamicin (9 d)<br>Vancomycin (10 d)
2	Non-LRE	9	*Klebsiella pneumoniae*	CSF	No growth	Leukocytosis on routine check prompting evaluation	CLABSI	Gentamicin (3 d)<br>Vancomycin (2 d)<br>Cefepime (10 d)
3	Non-LRE	29	*Streptococcus agalactiae*	CSF	No growth	Lethargy with abdominal distention and emesis	BSI with ileus	Gentamicin (4 d)<br>Vancomycin (2 d)<br>Piperacillin-tazobactum (11 d)
4	Non-LRE	14	*Serratia marcescens*	CSF	No growth	Increased apnea events	CLABSI, meningitis with cerebral abscesses	Gentamicin (6 d)<br>Vancomycin (1 d)<br>Cefepime (37 d)<br>Meropenam (11d)<br>Piperacillin-tazobactum (2 d)
5	Non-LRE	14	*K. pneumoniae ssp pneumoniae*	None	N/A	Increased abdominal distension and emesis	BSI/NEC	Piperacillin-tazobactum (4d)<br>Vacomycin (3d)<br>Gentamycin (1 d)<br>Cefepime (20 d)<br>Metronidazole (21 d)
6	Non-LRE	11	*Escherichia coli*	CSF	No growth	Increased apnea events	BSI	Gentamicin (3 d)<br>Vancomycin (1 d)<br>Cefepime (3 d)<br>Ceftazadime (8 d)
7	Non-LRE	30	*E. coli*	CSF and urine	No growth	Increased apnea events	BSI and NEC	Gentamicin (3 d)<br>Vancomycin (9 d)<br>Cefepime (4 d)

								Ceftazadime (1 d)<br>Piperacillin-tazobactum (11 d)
8	Non-LRE	16	*Staphylococcus aureus*	Abscess and CSF	*S. aureus* (abscess); No growth (CSF)	Increased oxygen requirement and mass on flank concerning for abscess	CLABSI with abscess	Vancomycin (6 d)<br>Gentamicin (3 d)<br>Oxacillin (28 d)<br>Rifampin (4 d)<br>Cefipime (2 d)<br>Piperacillin-tazobactum (8 d)
9	Non-LRE	11	*E. coli* and *S. epidermidis*	Tracheal aspirate	*E. coli*	Increased oxygen or respiratory support	CLABSI, could not rule out meningitis	Gentamicin (6 d)<br>Vancomycin (10 d)<br>Cefepime (23 d)
**LOS evaluations with negative cultures managed without extended antibacterial treatment**								
10	LRE	7	No growth	None	N/A	Abdominal symptoms: emesis and discoloration	Not infected	Gentamicin (1 d)<br>Vancomycin (3 d)
11	LRE	7	No growth	None	N/A	Hyperglycemia after manipulation for procedure; increased apnea events	Not infected	Gentamicin (2 d)<br>Vancomycin (3 d)
12	Non-LRE	33	No growth	Urine and viral panel	No growth	Increased oxygen or respiratory support	Postnatal cytomegalovirus infection	Gentamicin (2 d)<br>Vancomycin (3 d)
13	LRE	6	No growth	None	N/A	Increased bands on routine complete blood count	Not infected	Gentamicin (1 d)<br>Vancomycin (2 d)
14	LRE	28	No growth	Tracheal aspirate and urine	CoNS (tracheal aspirate); No growth (urine)	Increased oxygen or respiratory support and leukopenia on complete blood count	Not infected	Gentamicin (2 d)<br>Oxacillin (3 d)
15	Non-LRE	13	No growth	Urine	No growth	Increased apnea events	Not infected	Gentamicin (2 d)<br>Vancomycin (3 d)
16	Non-LRE	14	No growth	None	N/A	Increased oxygen or respiratory support	Not infected	Gentamicin (2 d)<br>Vancomycin (2 d)

Footnotes: [1]Sites for blood culture for episodes of bacteremia are as follows – 1) central line, 2) both central line and peripheral, 3) peripheral, 4) peripheral, 5) peripheral, 6) peripheral, 7) peripheral, 8) central line and peripheral, and 9) peripheral. All LOS evaluations with negative results were peripheral cultures except episode 16 which was a central line culture. [2]All antibiotic courses were started after blood culture was obtained; different antibiotics do not always

start on the same day. Abbreviations – *BSI*, blood stream infection; *CLABSI*, central line-associated blood stream infection; *CoNS*, coagulase-negative staphylococci; *CSF*, cerebrospinal fluid; *DOL*, day of life; *LRE*, low-risk for early-onset sepsis; *LOS*, late-onset sepsis; *N/A*, not applicable; *NEC*, necrotizing enterocolitis.

Table S2. Number of samples and patients included in the microbiome analysis for each time period

Hours after birth	No. of samples	No. of patients	Median samples per patient, (IQR)
0 to ≤ 72 hours (Days 1-3)	22	17	0 (0-1)
73 to ≤ 168 hours (Days 4-7)	76	40	1 (1-2)
169 to ≤ 336 hours (Week 2)	66	46	1 (1-2)
337 to ≤ 504 hours (Week 3)	47	42	1 (1-2)
505 to ≤ 672 hours (Week 4)	41	41	1 (1-2)
673 to ≤ 840 hours (Week 5)	49	43	1 (1-2)
841 to ≤ 1080 hours (Week 6)	54	41	1 (1-2)
≥1081 hours (Week 7)	5	5	0 (0-0)
Total	360	48	7 (7-9)

Figure S1. Diet characteristics in study cohort. (a) Cumulative incidence of infants receiving antibiotics during hospitalization. (b) Timelines depicting the number of study infants to whom the corresponding antibiotics were administered. (c) Number of infants administered mother's milk, donor milk, and formula throughout the study. (d) Percentage of infants who were nil per os in each day of life. (e) Timeline of diet and fortification. Each square of the grid represents one day in the life of an infant enrolled in the study. Abbreviations – *LRE*, low-risk for early-onset sepsis.

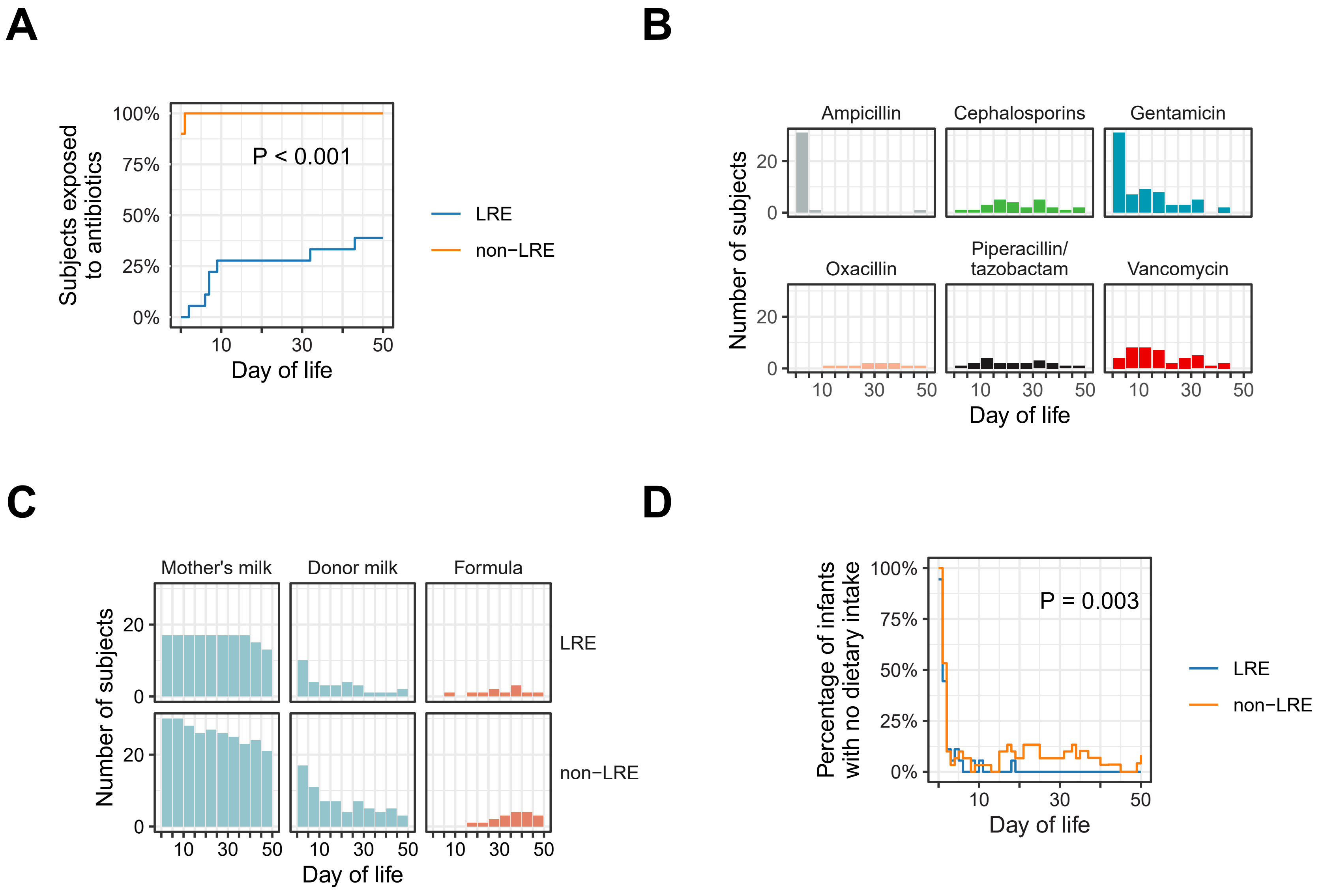

A
Subjects exposed to antibiotics
100%
75%
50%
25%
0%
P < 0.001
10
30
50
Day of life
LRE
non−LRE
B
Ampicillin
Cephalosporins
Gentamicin
Oxacillin
Piperacillin/ tazobactam
Vancomycin
Number of subjects
20
0
Day of life
C
Mother's milk
Donor milk
Formula
LRE
non−LRE
Number of subjects
Day of life
D
Percentage of infants with no dietary intake
P = 0.003
Day of life
LRE
non−LRE

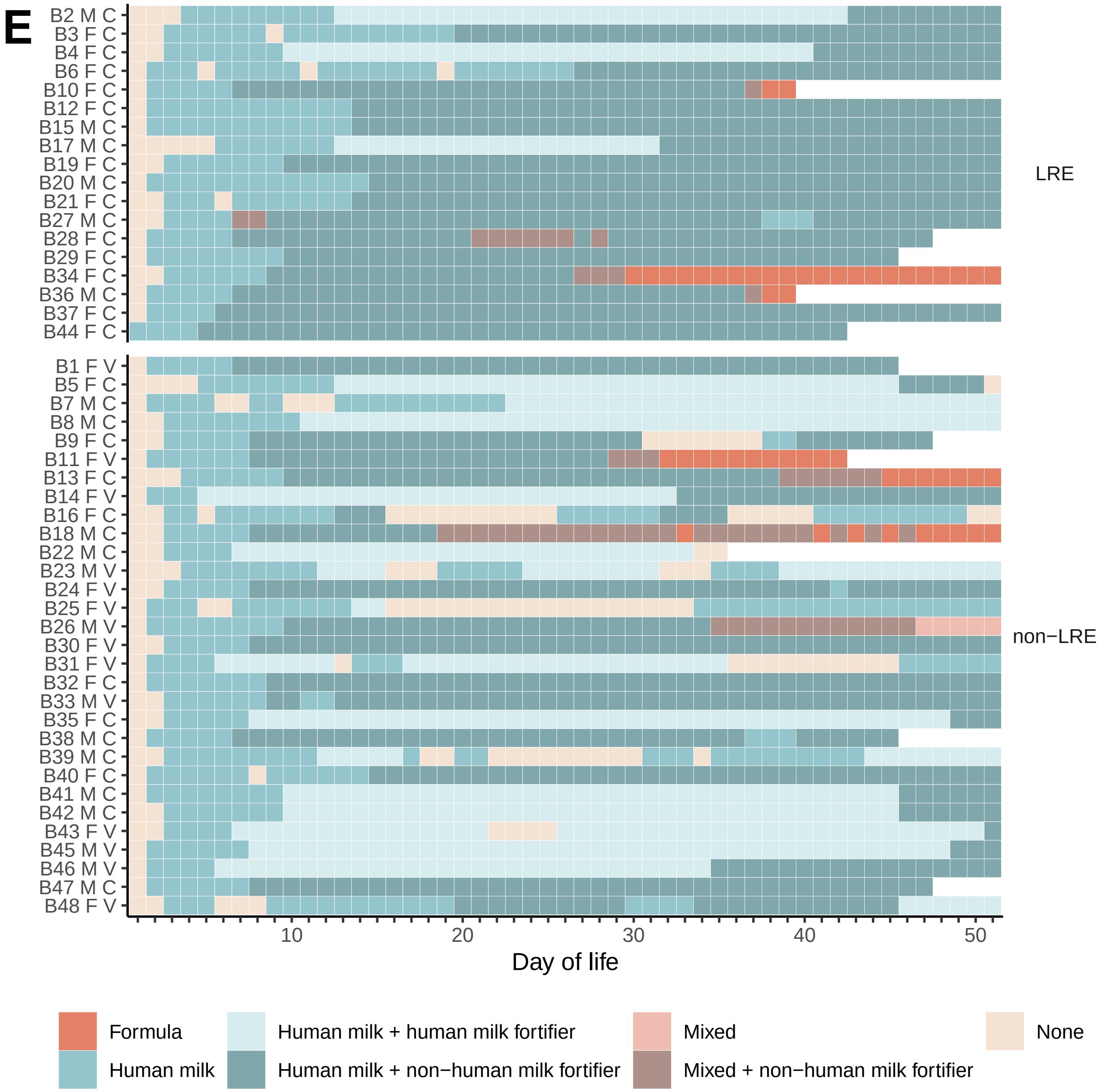

E
B2 M C
B3 F C
B4 F C
B6 F C
B10 F C
B12 F C
B15 M C
B17 M C
B19 F C
B20 M C
B21 F C
B27 M C
B28 F C
B29 F C
B34 F C
B36 M C
B37 F C
B44 F C
LRE
B1 F V
B5 F C
B7 M C
B8 M C
B9 F C
B11 F V
B13 F C
B14 F V
B16 F C
B18 M C
B22 M C
B23 M V
B24 F V
B25 F V
B26 M V
B30 F V
B31 F V
B32 F C
B33 M V
B35 F C
B38 M C
B39 M C
B40 F C
B41 M C
B42 M C
B43 F V
B45 M V
B46 M V
B47 M C
B48 F V
non−LRE
10
20
30
40
50
Day of life
Formula
Human milk
Human milk + human milk fortifier
Human milk + non−human milk fortifier
Mixed
Mixed + non−human milk fortifier
None

Figure S2. Bacterial community composition during the first week of life. All samples from the first week were subjected to Principal Coordinates Analysis using Bray-Curtis distance. Samples from days 1-3 (0-72h) are shown separately from those obtained on days 4-7 (72-168h) of life. Abbreviations – *LRE*, low-risk for early-onset sepsis.

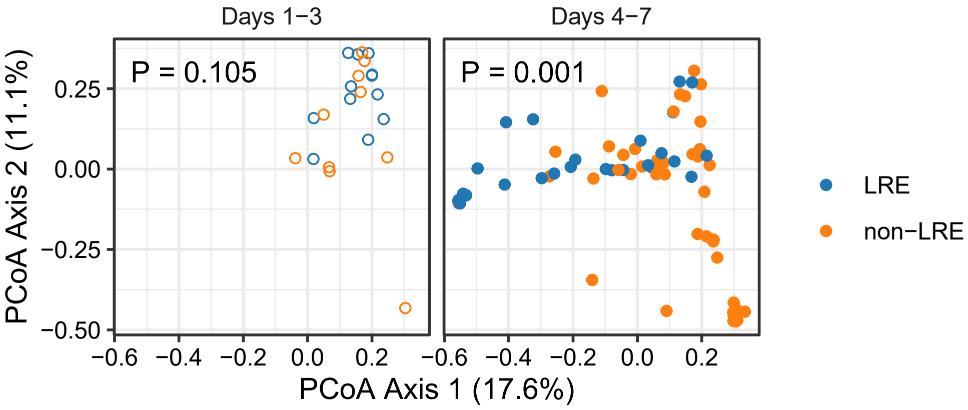

Days 1−3
Days 4−7
P = 0.105
P = 0.001
PCoA Axis 2 (11.1%)
PCoA Axis 1 (17.6%)
0.25
0.00
−0.25
−0.50
−0.6
−0.4
−0.2
0.0
0.2
LRE
non−LRE

Figure S3. Bacterial community composition and species relative abundance after the first week of life. (a) Bray-Curtis distance between samples collected after the first week of life. (b) Bacterial species found to be increasing more rapidly in LRE (*Citrobacter freundii*, *Enterobacter cloacae*, and *Escherichia coli*) or non-LRE infants (*Enterococcus faecalis*) after the first week of life. (c) Bacterial species found to be increasing or decreasing in abundance with infant age in both groups but were not different between LRE and non-LRE infants. Abbreviations – *LRE*, low-risk for early-onset sepsis.

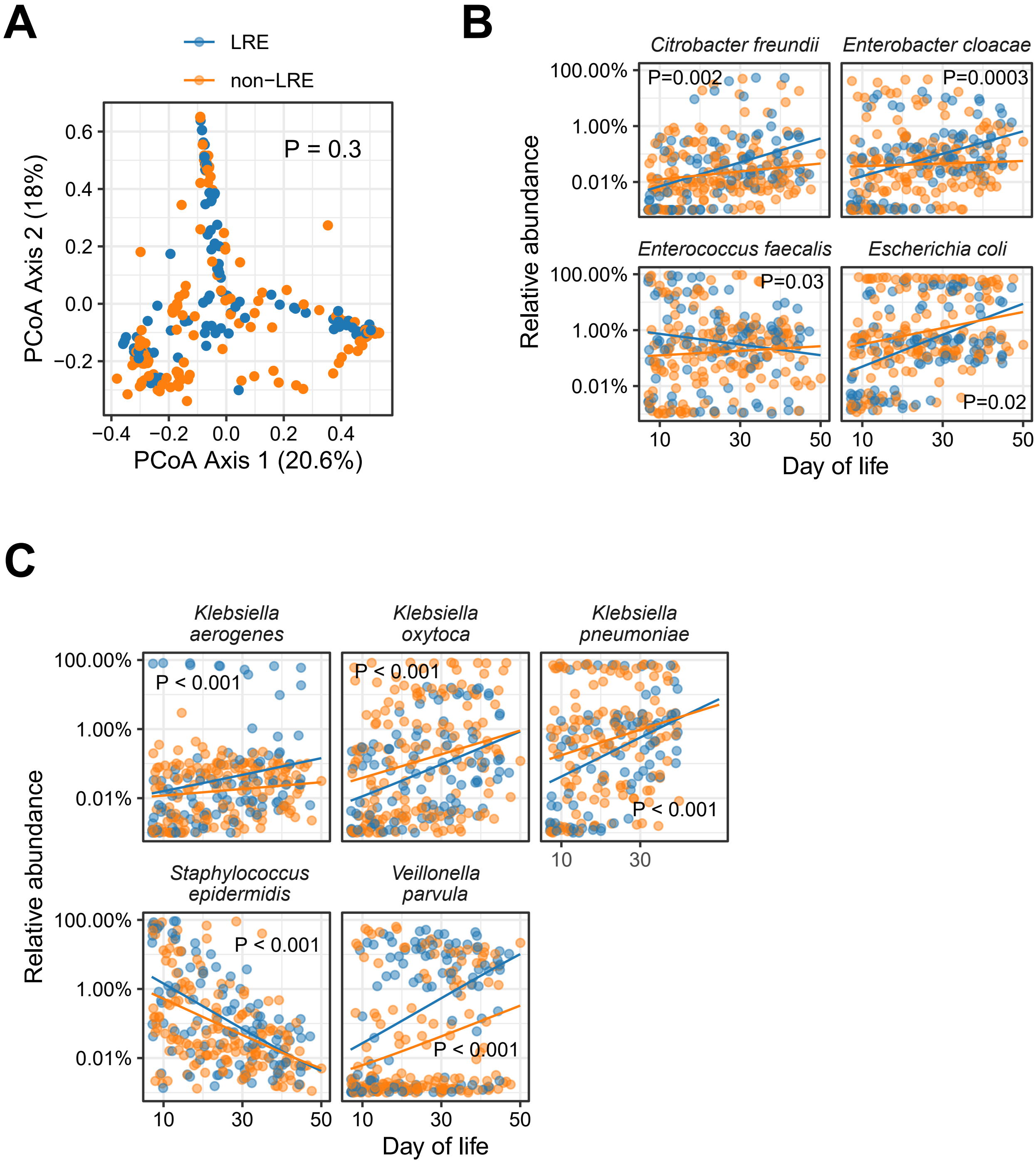
A
LRE
non−LRE
P = 0.3
PCoA Axis 2 (18%)
PCoA Axis 1 (20.6%)
B
Citrobacter freundii
P=0.002
Enterobacter cloacae
P=0.0003
Enterococcus faecalis
P=0.03
Escherichia coli
P=0.02
Relative abundance
Day of life
C
Klebsiella aerogenes
P < 0.001
Klebsiella oxytoca
P < 0.001
Klebsiella pneumoniae
P < 0.001
Staphylococcus epidermidis
P < 0.001
Veillonella parvula
P < 0.001
Relative abundance
Day of life

Figure S4. Abundance of virulence factors in the microbiome of LRE vs. non-LRE infants. Gene orthologs are shown if one or more time points differed between LRE and non-LRE groups. In each plot, the x-axis has units of reads per kilobase gene length per million reads (RPKM) in each sample. Lines extend from the first to the third quartile, and the median is indicated by a point. An asterisk indicates a difference in gene abundance between LRE and non-LRE infants after controlling for a 5% false discovery rate. Abbreviations – *LRE*, low-risk for early-onset sepsis.

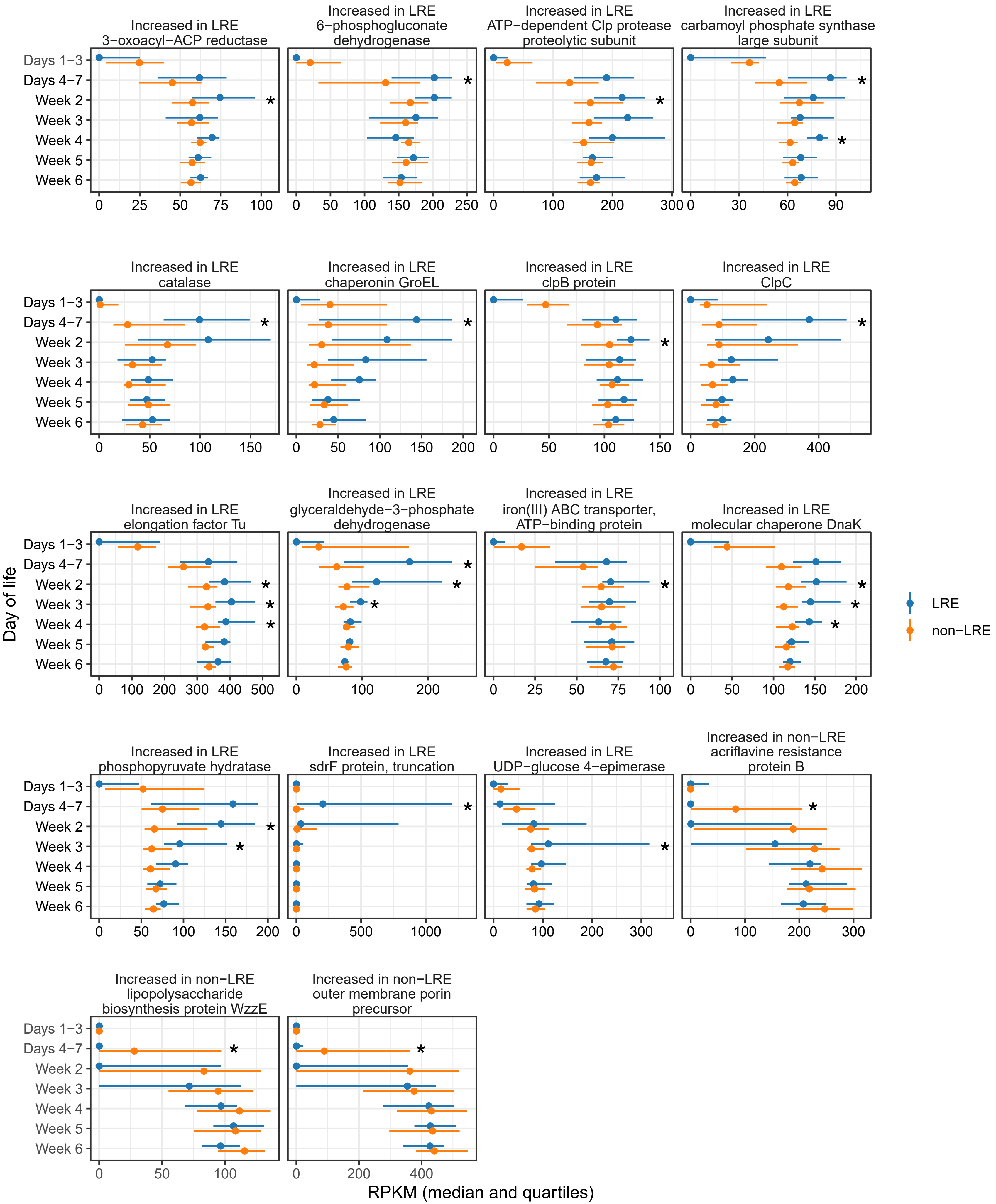

Increased in LRE 3−oxoacyl−ACP reductase
Increased in LRE 6−phosphogluconate dehydrogenase
Increased in LRE ATP−dependent Clp protease proteolytic subunit
Increased in LRE carbamoyl phosphate synthase large subunit
Increased in LRE catalase
Increased in LRE chaperonin GroEL
Increased in LRE clpB protein
Increased in LRE ClpC
Increased in LRE elongation factor Tu
Increased in LRE glyceraldehyde−3−phosphate dehydrogenase
Increased in LRE iron(III) ABC transporter, ATP−binding protein
Increased in LRE molecular chaperone DnaK
Increased in LRE phosphopyruvate hydratase
Increased in LRE sdrF protein, truncation
Increased in LRE UDP−glucose 4−epimerase
Increased in non−LRE acriflavine resistance protein B
Increased in non−LRE lipopolysaccharide biosynthesis protein WzzE
Increased in non−LRE outer membrane porin precursor
Days 1−3
Days 4−7
Week 2
Week 3
Week 4
Week 5
Week 6
Day of life
RPKM (median and quartiles)
LRE
non−LRE

Figure S5. Abundance of antibiotic resistance genes in the microbiome of LRE vs. non-LRE infants. Gene orthologs are shown if one or more time points differed between LRE and non-LRE groups. In each plot, the x-axis has units of reads per kilobase gene length per million reads (RPKM) in each sample. Lines extend from the first to the third quartile, and the median is indicated by a point. An asterisk indicates a difference in gene abundance between LRE and non-LRE infants after controlling for a 5% false discovery rate. Abbreviations – *LRE*, low-risk for early-onset sepsis.

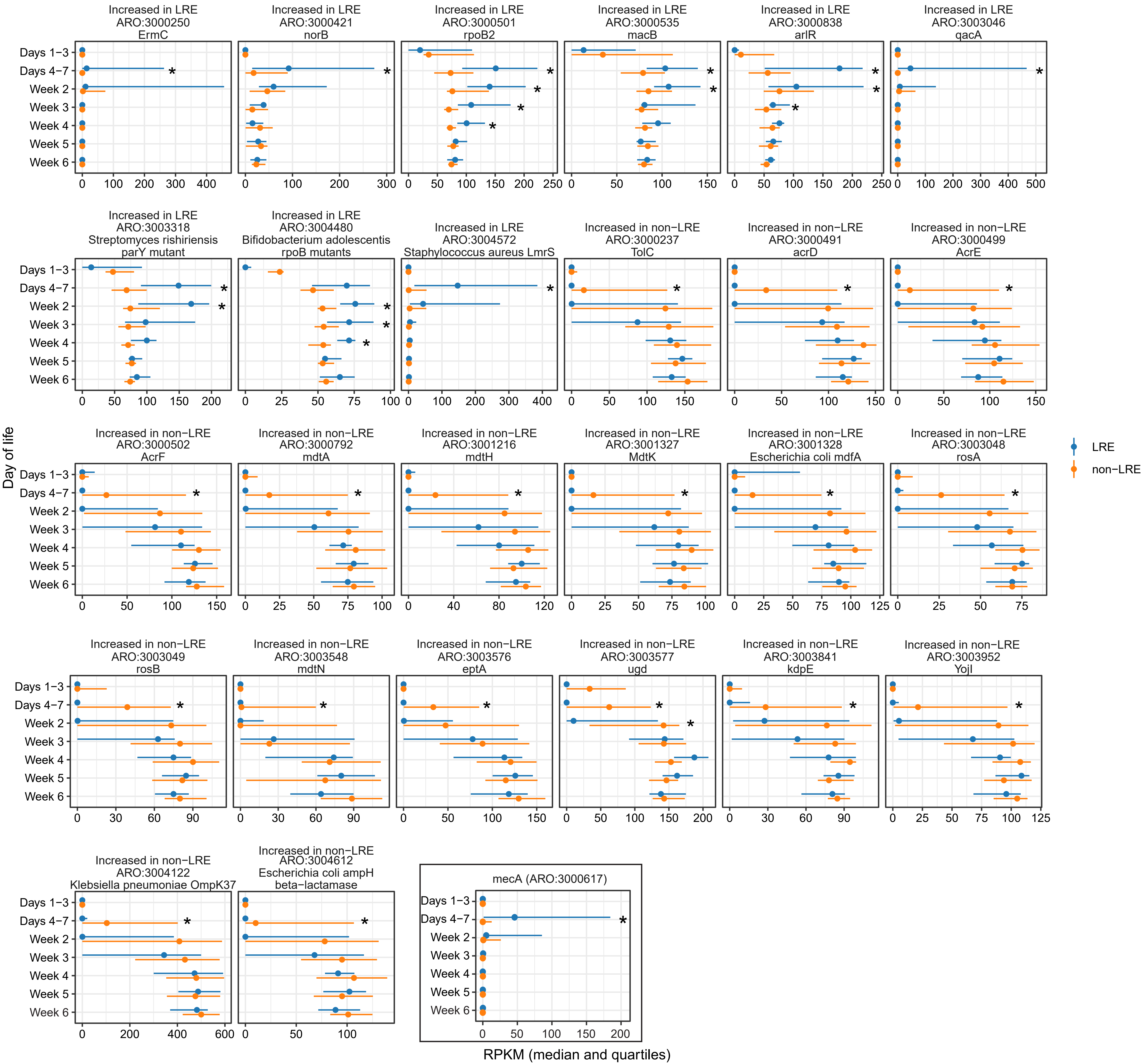
Increased in LRE ARO:3000250 ErmC
Increased in LRE ARO:3000421 norB
Increased in LRE ARO:3000501 rpoB2
Increased in LRE ARO:3000535 macB
Increased in LRE ARO:3000838 arlR
Increased in LRE ARO:3003046 qacA
Increased in LRE ARO:3003318 Streptomyces rishiriensis parY mutant
Increased in LRE ARO:3004480 Bifidobacterium adolescentis rpoB mutants
Increased in LRE ARO:3004572 Staphylococcus aureus LmrS
Increased in non−LRE ARO:3000237 TolC
Increased in non−LRE ARO:3000491 acrD
Increased in non−LRE ARO:3000499 AcrE
Increased in non−LRE ARO:3000502 AcrF
Increased in non−LRE ARO:3000792 mdtA
Increased in non−LRE ARO:3001216 mdtH
Increased in non−LRE ARO:3001327 MdtK
Increased in non−LRE ARO:3001328 Escherichia coli mdfA
Increased in non−LRE ARO:3003048 rosA
Increased in non−LRE ARO:3003049 rosB
Increased in non−LRE ARO:3003548 mdtN
Increased in non−LRE ARO:3003576 eptA
Increased in non−LRE ARO:3003577 ugd
Increased in non−LRE ARO:3003841 kdpE
Increased in non−LRE ARO:3003952 YojI
Increased in non−LRE ARO:3004122 Klebsiella pneumoniae OmpK37
Increased in non−LRE ARO:3004612 Escherichia coli ampH beta−lactamase
mecA (ARO:3000617)
Days 1−3
Days 4−7
Week 2
Week 3
Week 4
Week 5
Week 6
Day of life
LRE
non−LRE
RPKM (median and quartiles)

Figure S6. Microbiome characteristics in pre-antibiotic samples obtained prior to episodes of bacteremia compared to episodes with negative evaluations. (a) Timeline of diet, antibiotics, clinical culture results, and sample collection for episodes of bacteremia. Each square represents a day in the life of the child with the episode. A filled black dot (•) denotes a fecal sample, a large open circle (◯) denotes a negative blood culture, a diagonal cross denotes (╳) denotes a positive blood culture, and colored lines above the boxes indicate the type of antibiotics administered. (b) Relative abundance of bacterial species in the pre-antibiotic sample from each episode. Points are highlighted if the species was obtained from blood culture in that episode. (c) Timeline of diet, antibiotics, clinical culture results, and sample collection for episodes of suspected bacteremia evaluations that resulted in negative cultures. (d) Shannon diversity and the number of observed bacterial species in pre-antibiotic specimens between episodes that resulted in a diagnosis of bacteremia versus those with negative evaluations. (e) Detection of species causing bacteremia in pre-antibiotic samples from infants with subsequent bacteremia and from pre-antibiotic samples from infants with a negative evaluation.

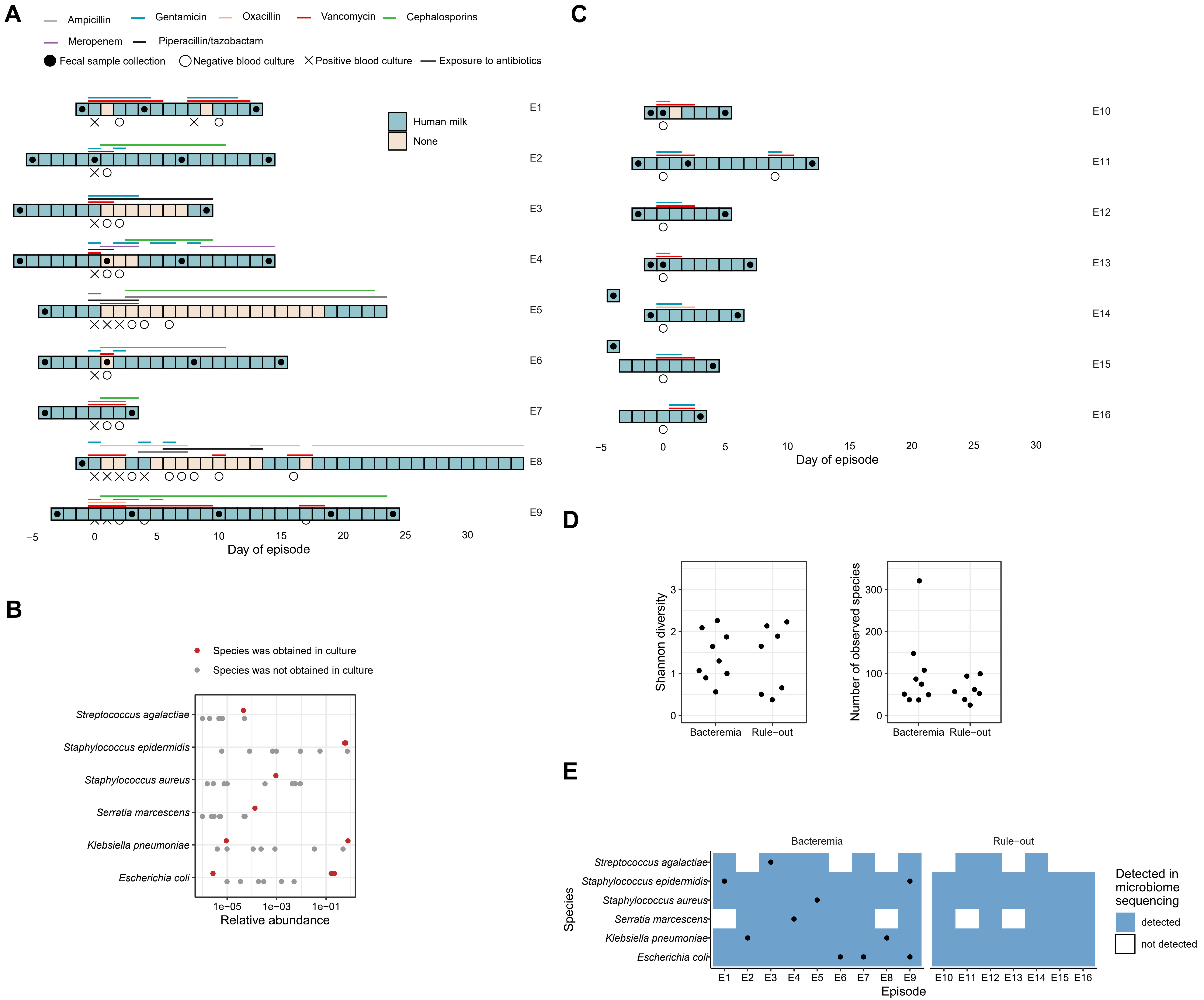

A
Ampicillin
Gentamicin
Oxacillin
Vancomycin
Cephalosporins
Meropenem
Piperacillin/tazobactam
Fecal sample collection
Negative blood culture
Positive blood culture
Exposure to antibiotics
Human milk
None
E1
E2
E3
E4
E5
E6
E7
E8
E9
Day of episode
C
E10
E11
E12
E13
E14
E15
E16
Day of episode
B
Species was obtained in culture
Species was not obtained in culture
Streptococcus agalactiae
Staphylococcus epidermidis
Staphylococcus aureus
Serratia marcescens
Klebsiella pneumoniae
Escherichia coli
Relative abundance
D
Shannon diversity
Number of observed species
Bacteremia
Rule−out
E
Species
Episode
Detected in microbiome sequencing
detected
not detected
